# Dendritic Cell-Based Immunotherapy for Prostate Cancer

**DOI:** 10.1155/2010/517493

**Published:** 2010-11-04

**Authors:** Hanka Jähnisch, Susanne Füssel, Andrea Kiessling, Rebekka Wehner, Stefan Zastrow, Michael Bachmann, Ernst Peter Rieber, Manfred P. Wirth, Marc Schmitz

**Affiliations:** ^1^Institute of Immunology, Medical Faculty, Technical University of Dresden, Fetscherstr. 74, 01307 Dresden, Germany; ^2^Department of Urology, Medical Faculty, Technical University of Dresden, 01307 Dresden, Germany; ^3^Translational Sciences and Safety, Novartis Biologic, 4002 Basel, Switzerland; ^4^Center for Regenerative Therapies Dresden, 01307 Dresden, Germany

## Abstract

Dendritic cells (DCs) are professional antigen-presenting cells (APCs), which display an extraordinary capacity to induce, sustain, and regulate T-cell responses providing the opportunity of DC-based cancer vaccination strategies. Thus, clinical trials enrolling prostate cancer patients were conducted, which were based on the administration of DCs loaded with tumor-associated antigens. These clinical trials revealed that DC-based immunotherapeutic strategies represent safe and feasible concepts for the induction of immunological and clinical responses in prostate cancer patients. In this context, the administration of the vaccine sipuleucel-T consisting of autologous peripheral blood mononuclear cells including APCs, which were pre-exposed *in vitro* to the fusion protein PA2024, resulted in a prolonged overall survival among patients with metastatic castration-resistent prostate cancer. In April 2010, sipuleucel-T was approved by the United States Food and Drug Administration for prostate cancer therapy.

## 1. Introduction

Prostate cancer (PCa) represents the most common noncutaneous cancer and the second leading cause of cancer-related deaths in the United States with an estimated incidence of 192,280 cases and an estimated number of 27,360 deaths in 2009 [1]. In Europe, PCa is also the most frequent cancer diagnosed in men with an estimated number of 345,900 cases in 2006 [2]. Most of the patients are diagnosed with organ-confined disease, for which radical prostatectomy, radiotherapy, and brachytherapy are effective treatment modalities [3, 4]. Active surveillance, which includes active monitoring of the disease and start of treatment at pre-defined thresholds for progression, represents an alternative since most of these tumors would never become of vital clinical importance if they had not been detected. Although the majority of patients are successfully treated with radical prostatectomy or radiation therapy, approximately 30% of patients develop recurrent disease [5].

Androgen deprivation represents an effective treatment modality for recurrent PCa [3, 4]. Bisphosphonates can increase bone mineral density and reduce the risk of bone fractures, which are typical side effects of androgen deprivation therapy [6]. Similar effects are expected for the treatment with denosumab, a monoclonal antibody against receptor activator of NF-kappaB ligand, which acts as a key mediator for osteoclast function, activation, and survival [7].

Therapeutic options for patients with progressive disease under androgen deprivation therapy comprise secondary hormonal manipulation and nonhormonal therapy such as chemotherapy [3]. In the management of metastatic hormone-refractory PCa (HRPC), chemotherapy with docetaxel serves as reference treatment due to the demonstrated significant survival benefit [8, 9]. In patients with HRPC, bisphosphonates are useful for the treatment of skeletal complications and pain relief thereby improving quality of life and also providing a suitable medication for palliative care [3].

Despite of the therapeutic benefit of these approaches and the achieved prolongation of overall survival, additional treatment strategies are needed to prevent progression from localized to advanced disease and to further improve survival outcomes for patients with advanced PCa.

## 2. The Important Role of Dendritic Cells in Antitumor Immunity

Dendritic cells (DCs) are professional antigen-presenting cells (APCs), which display a unique capacity to induce, sustain, and regulate T-cell responses [10, 11]. In tumor setting, DCs circulate through the blood and migrate to tumor tissues, where they interact with malignant cells. Immature DCs are particularly efficient in the uptake of tumor-derived material. DC maturation is induced by tumor-derived molecules such as heat shock proteins and high-mobility-group box 1 protein as well as proinflammatory cytokines produced by various tumor-infiltrating immune cells. During maturation DCs migrate from tumor tissues to T-cell-rich areas of secondary lymphoid organs, where they activate tumor-reactive CD8^+^ cytotoxic T lymphocytes (CTLs) and CD4^+^ T cells. CD8^+^ CTLs efficiently recognize and destroy tumor cells, which expose peptides derived from tumor-associated antigens (TAAs) in the complex with human leukocyte antigen (HLA) class I molecules [12]. Clinical studies focusing on the adoptive transfer of cytotoxic effector cells revealed tumor regression in cancer patients [13]. CD4^+^ T cells recognizing peptides in the context of HLA class II molecules also play an important role in antitumor immunity [14]. CD4^+^ T cells improve the capacity of DCs to induce CTLs by the interaction between CD40 on DCs and CD40 ligand on activated CD4^+^ T cells. In addition, CD4^+^ T cells provide help for the maintenance and expansion of CTLs by secreting cytokines such as interleukin (IL)-2 and can eradicate tumor cells directly. Besides their extraordinary capacity to induce and stimulate T-cell responses, DCs efficiently improve the immunomodulatory and cytotoxic potential of natural killer cells, which essentially contribute to the elimination of tumor cells [15–17]. Furthermore, DCs can also directly mediate tumor-directed cytotoxicity [18–20]. Owing to their various antitumor effects, DCs evolved as promising candidates for vaccination protocols in cancer therapy [21, 22].

## 3. Prostate Cancer-Associated Antigens for DC-Based Immunotherapy

Based on the crucial role of T cells in the elimination of tumor cells, much attention has been paied to the identification of tumor-associated proteins, that may provide targets of tumor-reactive T cells, and on the definition of peptide motifs within these proteins serving as T-cell epitopes. Here, we focus on PCa-associated target antigens, which have already been used for DC-based vaccination trials enrolling PCa patients. A summary of these CD8^+^ T cell epitopes is demonstrated in Table 1.

Prostate-specific antigen (PSA), a kallikrein-like serin-protease, is almost exclusively expressed by prostate epithelial cells, can be detected in the majority of PCa tissues, and represents the most widely used serum marker for diagnosis and monitoring of PCa [39–42]. The identification of HLA-A2-restricted PSA-derived peptides was driven by *in vitro* approaches using peptide-pulsed or RNA-transfected APCs to activate tumor-reactive CTLs [23–25, 27, 43]. By combining several previously identified and novel PSA peptides in an oligopeptide, Correale et al. demonstrated the possibility of simultaneous induction of CTLs specific for different epitopes dependent on the HLA repertoire of the patient [26]. 

The integral membrane glycoprotein prostate-specific membrane antigen (PSMA) represents a marker for normal prostate cells and can be detected in the majority of prostate tumors, particularly in undifferentiated, metastatic HRPC [44, 45]. Several HLA-A2-restricted peptides were shown to induce tumor-reactive CTL responses *in vitro *and *in vivo *[28, 29]. 

Prostatic acid phosphatase (PAP) is a glycoprotein with enzymatic activity, which can be mainly detected in prostate tissue [46]. Peshwa et al. identified an HLA-A2-binding, endogenously generated, immunogenic peptide that induced tumor-directed CTLs *in vitro* [30]. 

Prostate stem cell antigen (PSCA) is a glycosylphosphatidylinositol-anchored cell surface glycoprotein, that is mainly expressed in the prostate [47]. PSCA expression is detectable in more than 80% of primary PCa samples and bone metastases. It is increased in both androgen-dependent and -independent prostate tumors when compared to the corresponding normal prostate tissues, particularly in carcinomas of high stages and Gleason Scores [47, 48]. We and others identified an HLA-A2-restricted PSCA peptide, which induced tumor-reactive CTL responses *in vitro* [31, 32]. Increased frequencies of CD8^+^ T cells specific for this peptide were found in the blood of PCa patients indicating the relevance of this epitope *in vivo* [32]. 

Prostein represents a transmembrane protein of the Golgi with unique specificity for normal and malignant prostate tissues [49, 50]. Our group found abundant expression in malignant and normal prostate tissues and maintained or even elevated transcript levels in 87% of the primary tumors compared to autologous nonmalignant tissue samples [33]. By *in vitro* stimulation of CD8^+^ T lymphocytes with peptide-loaded DCs, we identified an autochthonously generated, HLA-A2-presented peptide, that was capable of activating tumor-reactive CTLs [33]. 

The gene transient receptor potential (trp)-p8 encodes a seven-span transmembrane protein with significant homology to a family of Ca^2+^ channel proteins [51]. Trp-p8 is mainly detected in the prostate and shows an overexpression in PCa of early stages and low grades [34, 51]. We identified an HLA-A2-binding peptide, which is able to stimulate tumor-reactive CTLs *in vitro* [34]. 

Potential target structures, which are overexpressed in tumors of different origin including PCa comprise the human telomerase reverse transcriptase (hTERT), which is the catalytic subunit of telomerase, and survivin. hTERT is undetectable in most nontransformed somatic cells but is expressed in more than 85% of human tumors including PCa [52]. Several naturally generated CTL epitopes efficiently inducing peptide-specific and tumor-reactive CTLs *in vitro* and *in vivo* have been described. Thus, the generation of HLA-A2-restricted hTERT peptide-specific CTLs, which are able to lyse hTERT-expressing tumor cells of diverse histological origin including PCa cells has been reported [35, 36]. In addition, the peptide proved to be immunogenic *in vivo*, since immunization of HLA-A2.1 transgenic mice generated a specific CTL response [36]. Immunogenicity in mice could be markedly increased by an amino acid substitution at an HLA anchoring position [53]. 

Survivin, an inhibitor of apoptosis, is highly overexpressed in many human tumors including PCa, and its expression correlates with aggressiveness and poor prognosis of tumor disease [54, 55]. The wide expression in cancer and the functional role for tumor cell survival make survivin a promising target for T-cell-based immunotherapy. We described an endogenously produced HLA-A2-restricted peptide, which induced specific CTL responses *in vitro* [37]. Specific T-cell reactivity against this peptide motif was detected in the peripheral blood of chronic lymphatic leukemia patients and in tumor-infiltrated lymph nodes of melanoma patients [38].

## 4. Dendritic Cell-Based Immunotherapy for Prostate Cancer

**Figure 1:**
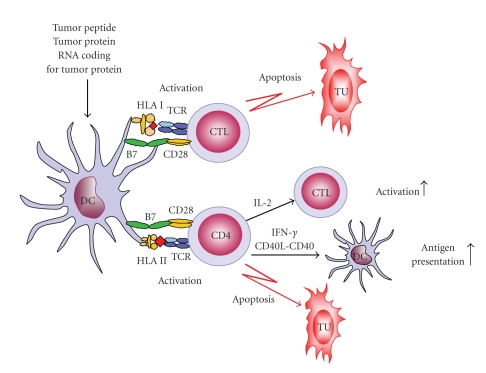
DC-based immunotherapeutic strategies for prostate cancer. DCs display a unique capacity to induce and maintain T-cell responses and emerged as promising candidates for vaccination strategies in prostate cancer therapy. Thus, DCs are loaded with PCa-associated antigen-derived peptides, protein, or RNA. Due to their high surface expression of HLA-peptide-complexes and costimulatory molecules, DCs efficiently activate and expand CD8^+^ CTLs and CD4^+^ T cells. CD8^+^ CTLs possess a profound capability to recognize and destroy tumor cells. CD4^+^ T cells enhance the capacity of DCs to induce CTLs by the interaction between CD40 on DCs and CD40 ligand on activated CD4^+^ T cells. In addition, they provide help for the maintenance and expansion of CTLs by secreting cytokines and are able to eradicate tumor cells directly. CTLs: cytotoxic T cells; DCs: dendritic cells; HLA: human leukocyte antigen; IL: interleukin; IFN: interferon; TCR: T cell receptor; TU: tumor cells.

DCs play a critical role for the induction of innate and adaptive antitumor immune responses. Due to their various antitumor effects, DCs emerged as attractive candidates for vaccination protocols in cancer therapy (Figure 1). Animal models demonstrated that TAA-presenting DCs are capable of inducing protective and therapeutic antitumor responses [56, 57]. Clinical trials enrolling B-cell lymphoma, melanoma, or renal cancer patients revealed promising immunologic and clinical responses of TAA-loaded DCs administered as a vaccine against cancer [58–61].

In prostate cancer setting, the administration of DCs pulsed with TAA-derived peptides was well tolerated and resulted in the induction of immunological and clinical responses in patients. Thus, a phase-I trial was initiated to evaluate the vaccination of DCs loaded with PSMA-derived peptides in HRPC patients [29, 62]. DCs were generated from monocytes in the presence of granulocyte-macrophage colony-stimulating factor (GM-CSF) and IL-4. Subsequently, the monocyte-derived DCs were pulsed with the HLA-A2-restricted PSMA-derived peptides PSM-P1 or PSM-P2. Nineteen patients received at least two infusions of up to 2 × 10^7^ peptide-loaded DCs at six- to eight-week intervals. Treatment was well tolerated except a mild to moderate transient hypotension. Five partial responders based on National Prostate Cancer Project criteria and a >50% reduction of PSA level were observed. Subsequently, a phase II trial was conducted to further investigate the therapeutic efficiency of PSMA peptide-pulsed DCs [63]. Six infusions of monocyte-derived DCs pulsed with PSM-P1 and PSM-P2 were administered at six week intervals. In addition, 17 patients received subcutaneous injections of GM-CSF. Nine partial responders based on National Prostate Cancer Project criteria and a >50% reduction of PSA level were identified in a group of 33 HRPC patients.

In another clinical study, monocyte-derived DCs pulsed with a hTERT-derived peptide and keyhole limpet hemocyanin were administered to five patients with metastatic HRPC [64]. DCs were subcutaneously injected every other week for up to six vaccinations. Peptide-reactive T cells were induced in two patients after vaccination. All four evaluable patients had stabilization of disease. Recently, we conducted a clinical study to evaluate the potential of DCs loaded with a cocktail consisting of HLA-A2-restricted peptides derived from PSA, PSMA, survivin, prostein, and trpp8 [65]. Immature DCs were generated from monocytes in the presence of GM-CSF and IL-4. For maturation, DCs were incubated with GM-CSF, IL-4, IL-1*β*, IL-6, tumor necrosis factor (TNF)-*α*, and prostaglandin E2. Subsequently, the mature monocyte-derived DCs were pulsed with five HLA-A*0201-restricted TAA-derived peptides. Eight HRPC patients received four vaccinations of every other week. Peptide-pulsed DCs were simultaneously injected intradermally and intravenously. One patient displayed a partial response. Three other patients showed stable disease over 4 to 17 weeks. Three of these four PSA responders exhibited specific T-cell responses against prostein, survivin, or PSMA. In another clinical trial, six HRPC patients were treated with mature monocyte-derived DCs pulsed with a cocktail consisting of HLA-A2-restricted peptides derived from PSA, PSCA, PSMA, and PAP [66]. Treatment was well tolerated. Three patients displayed specific T-cell responses against all antigens. Clinically, DC vaccination was associated with an increase in PSA doubling time. Thomas-Kaskel et al. initiated a clinical study to evaluate the vaccination of DCs pulsed with PSA- and PSMA-derived peptides in 12 patients with hormone- and chemotherapy-refractory PCa [67]. Patients received four vaccinations with a median of 2.7 × 10^7^ peptide-loaded mature monocyte-derived DCs subcutaneously in biweekly intervals. Six patients had stable disease and five patients developed delayed-type hypersensitivity (DTH) reactions. DTH-positivity was associated with superior survival. A significant correlation between DTH reactions and progression-free survival was not observed. Hildenbrand et al. conducted a clinical trial enrolling 12 HRCP patients, which was based on the combination of interferon (IFN)-*γ* and mature monocyte-derived DCs pulsed with three different HLA-A2-restricted PSA peptides [68]. Treatment consisted of the subcutaneous injection of IFN-*γ* followed by three intracutaneous administrations of 2 × 10^6^ peptide-loaded DCs. Vaccination was applied four times at three-week intervals. No severe side effects were observed. One patient displayed a partial response showing regression of lymph node metastases, four patients showed stable disease, one patient exhibited a mixed response, and six patients displayed progressive disease. 

Further clinical trials evaluated immunological and therapeutic efficiency of protein-loaded DCs in PCa patients. Thus, Fong et al. administered DCs loaded with recombinant murine PAP protein to 21 patients with metastatic PCa [69]. Patients received two injections monthly with a mean dose of 11,2 × 10^6^ cells per vaccination. Treatment was well tolerated. All patients developed T-cell immunity to mouse PAP and 11 patients to the homologous self-antigen human PAP. Six patients displayed clinical stabilization of their previously progressing PCa as determined by PSA level monitoring, computerized tomography, and bone scans. All these patients developed T-cell proliferation in response to human PAP. Another clinical trial was performed to evaluate the efficiency of mature monocyte-derived DCs pulsed with human recombinant PSA protein for the treatment of PCa patients in biochemical relapse after radical prostatectomy [70]. Twenty-four patients received nine administrations of PSA-loaded DCs by combined intravenous, subcutaneous, and intradermal routes over 21 weeks. No severe side effects were observed and 11 patients exhibited a transient PSA decrease. 

A particular promising immunotherapeutic strategy for advanced PCa patients is based on the administration of APCs pre-exposed *in vitro* to PA2024, a fusion protein consisting of human GM-CSF and PAP (APC8015, sipuleucel-T, Provenge). To generate sipuleucel-T, autologous peripheral blood mononuclear cells including APCs such as DCs were collected by two sequential buoyant density centrifugation steps and incubated with PA2024. Small et al. conducted sequential phase I and phase II trials including 31 HRPC patients to determine the safety and efficacy of sipuleucel-T [71]. Patients were treated intravenously with sipuleucel-T on weeks 0, 4, 8, and 24. Treatment was well tolerated. No patient had pre-existing T-cell responses or antibodies to PAP. After treatment, 38% of patients developed a T-cell response to PAP and 53% of patients had antibodies. Three patients had a more than 50% decline in PSA level and additional 3 patients displayed 25% to 49% decreases in PSA. In another phase II trial, 21 HRPC patients were vaccinated with sipuleucel-T [72]. In this study, the vaccine was administered intravenously twice, on weeks 0 and 2. The median number of cells was 2.7 × 10^9^ for the first infusion and 3.2 × 10^9^ for the second infusion. Subsequently, patients received three subcutaneous injections of PA2024 at weeks 4, 8, and 12. Two patients exhibited a 25% to 50% transient decrease in PSA level. For a third patient, PSA dropped to undetectable levels by week 24. The PSA level remained undetectable for 52 months and the metastatic adenopathy resolved. 

Rini et al. performed a clinical trial, which was based on the administration of sipuleucel-T and bevacizumab to 22 patients with recurrent PCa after definitive local therapy [73]. Bevacizumab is a recombinant antibody against vascular endothelial growth factor, that represents a proangiogenic protein with inhibitory effects on APCs. Patients received sipuleucel-T intravenously on weeks 0, 2, and 4 and bevacizumab on weeks 0, 2, and 4 and every two weeks thereafter until toxicity or disease progression were observed. Nine patients displayed a decrease of PSA, ranging from 6% to 72%. 

Following the results of the previous studies, a phase III study (D9901) enrolling 127 metastatic HRPC patients was conducted to determine the safety and therapeutic efficiency of sipuleucel-T in a placebo-controlled trial [74]. Patients were randomized to receive three infusions of the vaccine or placebo every two weeks with primary endpoint of time to disease progression. The median time to disease progression was not statistically significant at 11.7 weeks in the vaccine group compared with 10.0 weeks in the placebo group. However, a statistically significant increase in median overall survival was observed (25.9 months in the vaccine group; 21,4 months in the placebo group). 

More recently, Higano et al. performed an integral data analysis of the formerly described phase III study (D9901) and a second phase III trials (D9902A), which was also based on the administration of sipuleucel-T to HRPC patients [75]. Altogether, 225 patients were randomized to receive three infusions of sipuleucel-T (147 patients) or placebo (78 patients) every two weeks. Of the 147 patients in the sipuleucel-T arms, 5 patients showed a PSA reduction of >50% and two additional patients of >25%. Patients randomized to sipuleucel-T had a 21% reduction in the risk of disease progression and a 33% reduction in the risk of death compared with patients randomized to placebo. The median survival was of 23,2 months in the sipuleucel-T arms and 18,9 months in the placebo arms. The percentage of patients alive at 36 months was 33% in the sipuleucel-T arms and 15% in the placebo arms. Treatment was well tolerated. The overall incidence of adverse events was similar between patients treated with sipuleucel-T and patients treated with placebo. The most common adverse events were chills, pyrexia, headache, asthenia, dyspnea, vomiting, and tremor. Taken together, the integrated results of D9901 and D9902A demonstrate a survival benefit for patients treated with sipuleucel-T compared to patients treated with placebo. To further confirm the therapeutic efficiency of sipuleucel-T another randomised, placebo-controlled, multicenter phase III trial enrolling 512 patients with metastatic HRPC was conducted [76]. Patients on the sipuleucel-T treatment arm experienced a relative reduction of 22% in the risk of death compared with the placebo group. The median survival was 25,8 months in the sipuleucel-T group and 21,7 months in the placebo group. Based on these promising clinical results, the United States Food and Drug Administration recently approved sipuleucel-T for the treatment of asymptomatic or minimally symptomatic, metastatic HRPC. 

Further DC-based immunotherapeutic strategies for prostate cancer were evaluated in clinical trials. Thus, the potential of RNA-transfected DCs for PCa therapy was investigated. In this context, Heiser et al. vaccinated PSA RNA-transfected DCs to 13 metastatic PCa patients [77]. DCs were generated from monocytes in the presence of GM-CSF and IL-4 and subsequently transfected with PSA RNA. The PSA RNA-transfected DCs were administered at three escalating dose levels. Dose escalation was performed through an intravenous route with 1 × 10^7^, 3 × 10^7^, or 5 × 10^7^ cells applied at weeks 2, 4, and 6. For optimization, a concomitant dose of 1 × 10^7^ cells was given intradermally at each vaccination cycle. Induction of PSA-specific T-cell responses was found in all evaluated patients. Six of seven evaluated patients displayed a significant decrease in the log slope PSA. In another clinical trial, hTERT RNA-transfected DCs were administered to 20 metastatic PCa patients [78]. Vaccination resulted in an expansion of hTERT-specific T cells in 19 patients, was associated with a reduction of PSA velocity, and a molecular clearance of circulating tumor cells. Mu et al. conducted a clinical study enrolling 20 HRPC patients, which was based on the administration of monocyte-derived DCs transfected with RNA from allogeneic PCa cell lines [79]. Each patient received at least four weekly injections with 2 × 10^7^ transfected DCs either intranodally or intradermally. Thirteen of 19 patients that completed vaccination displayed a decrease in log slope PSA. 

Another vaccination strategy for prostate cancer is based on the administration of c-fms-like tyrosine kinase 3 (Flt3) ligand. This immunostimulatory agent efficiently promotes the differentiation and expansion of DCs *in vitro* and *in vivo*. Higano et al. conducted a trial evaluating the efficiency of Flt3 ligand in HRPC patients [80]. Treatment was well tolerated. Flt3 ligand application resulted in a marked increase of DC number in the peripheral blood. Eleven of 31 patients showed a decrease or only a minor increase (<25%) in PSA levels.

## 5. Conclusion

DCs play a crucial role for the induction of innate and adaptive antitumor immune responses. Thus, they efficiently activate and expand tumor-reactive CD8^+^ CTLs and CD4^+^ T cells. In addition, DCs can markedly improve the immunomodulatory and cytotoxic potential of natural killer cells and can directly mediate tumor-directed cytotoxicity. Due to their various antitumor effects, DCs emerged as promising candidates for the treatment of PCa patients. Consequently, several clinical trials enrolling PCa patients were conducted, which were based on the administration of DCs pulsed with TAA-derived peptides, protein, or RNA. These studies demonstrated that DC-based immunotherapeutic strategies represent safe and feasible concepts for the induction of immunological and clinical responses in PCa patients. Recently, sipuleucel-T consisting of PA2024 fusion protein-loaded APCs was approved by the United States Food and Drug Administration for the treatment of asymptomatic or minimally symptomatic, metastatic HRPC. Despite these promising clinical effects the efficiency of the various DC-based treatment modalities for many patients with advanced PCa is still limited. Therefore, further improvement is required, which may be achieved by combining DC-based vaccination strategies with antibody-, radio-, hormone-, chemo-, or antiangiogenic therapy.

## Figures and Tables

**Table 1:** PCa-associated antigen-derived CD8^+^ T-cell epitopes used for DC-based immunotherapy.

Antigen	HLA restriction element	Peptide position	Amino acid sequence	References
Prostate-specific antigen (PSA)	HLA-A2	146–154	KLQCVDLHV	[23, 24]
		141–150	FLTPKKLQCV	[25, 26]
		154–163	VISNDVCAQV	[25–27]
Prostate-specific membrane antigen (PSMA)	HLA-A2	4–12	LLHETDSAV	[28, 29]
		711–719	ALFDIESKV	[29]
Prostatic acid phosphatase (PAP)	HLA-A2	299–307	ALDVYNGLL	[30]
Prostate stem cell antigen (PSCA)	HLA-A2	14–22	ALQPGTALL	[31, 32]
Prostein	HLA-A2	31–39	CLAAGITYV	[33]
Transient receptor potential-p8 (Trp-p8)	HLA-A2	187–195	GLMKYIGEV	[34]
Human telomerase reverse transcriptase (hTERT)	HLA-A2	540–548	ILAKFLHWL	[35, 36]
Survivin	HLA-A2	95–104	ELTLGEFLKL	[37, 38]
